# Dietary sodium butyrate improves intestinal development and function by modulating the microbial community in broilers

**DOI:** 10.1371/journal.pone.0197762

**Published:** 2018-05-24

**Authors:** Wei Wu, Zhibin Xiao, Wenyi An, Yuanyang Dong, Bingkun Zhang

**Affiliations:** State Key Laboratory of Animal Nutrition, College of Animal Science and Technology, China Agricultural University, Beijing, P. R. China; Wageningen University, NETHERLANDS

## Abstract

This study investigated the effects of dietary sodium butyrate (SB) supplementation, provided as a specially coated product, on growth performance, intestinal development, morphological structure and function in broilers. In total, 720 one-day-old Arbor Acres male broilers were randomly allocated into six treatment groups with six replicates each and then fed basal diets (control) supplemented with 0, 200, 400, 800 or 1000 mg/kg of SB or with antibiotics (100 mg/kg aureomycin and 20 mg/kg colistin sulfate). The growth trial lasted for 42 days. No differences (*P*>0.05) in growth performance were detected between groups during the grower period (1–21 d) or over the total (1–42 d) trial period, whereas the addition of SB improved the intestinal structure by stimulating (*P*<0.05) goblet cells on jejunal and ileal villi accompanied by a trend towards increased (*P*_*diets*_<0.10) ileal villus height. In addition, more inerratic leaf-shaped villi and mucus secretion and significantly fewer erosions were demonstrated by scanning electron microscopy. Apart from decreased (*P*<0.05) malondialdehyde (MDA) in the ileal mucosa at 21 d of age, supplemental SB at higher doses (800 mg/kg) led to greater (*P*<0.05) total antioxidant capacity and depressed (*P*<0.05) MDA concentrations in the jejunal mucosa. Birds fed with 400 mg/kg and 800 mg/kg SB had higher (*P*<0.05) acetic acid concentrations at 42 d and higher butyric acid at 21 d in the jejunum chyme. Morever, chicks fed SB diet were found to have higher concentrations of butyric acid (*P*<0.05) in the ileal chyme. SB additions at 400 mg/kg displayed higher *Firmicutes* and *Proteobacteria* levels, while a higher (*P*<0.05) relative abundance of *Bacteroidetes* was observed at 800 mg/kg. Furthermore, we found a striking decrease in *Enterobacteriaceae* and increases in *Lachnospiraceae* and *Rikenellaceae* in the cecal lumen of birds fed 800 mg/kg SB as well as a higher proportion of *Ruminococcaceae* and a noticeable reduction (*P*<0.05) of *Lactobacillaceae* in birds treated with 400 mg/kg SB. Taken together, our results support the importance of SB in improving the intestinal development, morphological structure and biological functions of broilers through modulation of the microbial community, which seems to be optimized for gut health at higher doses (800 mg/kg) of SB.

## Introduction

Sub-therapeutic doses of antibiotics have been recognized as growth enhancers for hosts and were extensively employed in poultry production as essential additives until their indiscriminate use was questioned as a public health concern due to antimicrobial resistance and residues [1]. As a result, many nutrients or dietary supplements have been investigated as potential alternatives to maintain functions of the gut and immune system, among which organic acids (commonly known as acidifiers or related salts) are generally beneficial, suitable and approved for in-feed use. Short-chain fatty acids (SCFAs), primarily acetate, propionate and butyrate, are crucial microbial metabolites that originate from the fermentation of undigested carbohydrates or from minor parts of dietary and endogenous proteins in the caeca and colon of host animals [2,3]. Butyrate, in particular, can be transported preferentially and appears to be the preferred energy source for the ameliorative growth of normal epithelial cells [4–6]. Meanwhile, butyrate also carries multiple benefits for gut integrity and health by stimulating intestinal blood flow [7], mucin secretion [8], electrolyte and water absorption [9], zonula occluden-1 (ZO-1) expression [10] as well as histone deacetylase (HDAC) inhibitors, which suppress colorectal cancer via the regulation of cell proliferation, differentiation and apoptosis [11]. Moreover, much attention has been devoted to a broad range of biological functions of this molecule, including its ability to inhibit pathogenic bacteria, modify immune and inflammatory responses and alter antioxidant capacities [12–14]. Accordingly, butyrate is known as a safe alternative to antibiotics in feed [15] following the ban on the utilization of antibiotic growth promoters in the poultry feed industry enacted by the European Union in 2005.

Because of the unpleasant odor and potentially labile volatility of butyric acid, sodium butyrate (SB) has been typically applied in poultry production and is readily transformed into the effective component within the gastrointestinal tract of birds. Supplementation with SB is thought to improve development of the intestinal mucosa and morphological structures [15–17] as well as modulate the growth of symbiotic intestinal microbiota; thus, dietary SB may be conducive to the physiological function and health of the gut. As recently reported in specific pathogen-free male mice, SB played a pivotal role in restoring the dysbiosis of gut microbiota by elevating the abundances of *Lactobacillus*, *Blautia* and *Christensenellaceae*, which participated in a virtuous circle of butyric acid [10]. In addition, SB has potential anti-inflammatory and immune-enhancing properties [18], affecting the expression of inflammatory cytokines, such as IL-6, IL-8, IFN-γ, TGF-β and IL-1β [19]. Previous studies have demonstrated that the effects of SB on growth performance of broilers are variable and are either stimulative or not significant [20–22]. Moreover, the mechanism of the beneficial action of SB remains unclear. The microbiota has been proven to exert a vital role in host health, and there is interplay between dietary and gut microbiota. However, how and to what degree SB supplementation influences microbial composition and diversity in broiler chickens remains poorly understood. Hence, the current experiment was comprehensively designed to explore the effects of SB supplementation on growth performance, intestinal histomorphology and function as well as on the microbial community in broilers. We used an innovative SB product that was protected by its salt structure and remained insoluble and undissociated in the stomach pH, mostly releasing in the duodenum and proximal jejunum of the animal. Here, we provide evidence that sodium butyrate facilitates feed efficiency, gut development and health through ameliorating intestinal microbial community structures, which reflects a compelling set of links between the composition of the gut microbes, the host diet and intestinal physiology.

## Materials and methods

### Experimental design

All experimental design and procedures for this study were approved and conducted under the supervision of the China Agricultural University Animal Care and Use Committee, which has adopted the Animal Care and Use Guidelines governing all animals used in experimental procedures. In total, 720 one-day-old Arbor Acres male broilers were weighed and randomly allocated to six treatments with six replicates of 20 birds each so that their initial body weights were similar across all the groups. Chicks were raised in wire cages with nipple drinkers, and the house was controlled at standard conditions of temperature, humidity and ventilation and maintained on a 24-h constant-light regime throughout the trial period. The birds were fed with one of six diets: basal diets (control) and the experimental diets supplemented with SB at 200 (SB1), 400 (SB2), 800 (SB3), or 1000 (SB4) mg/kg or with antibiotics (100 mg/kg aureomycin+20 mg/kg colistin sulfate) for 42 days. A supplementation product (Butirex C4) that contained 54% sodium butyrate protected by a physical and chemical matrix of buffer salts was used in this study. The basal corn-soybean meal diets were formulated to meet or exceed the feeding standards of China (NY/T 2004) for broilers [23], whose composition and nutritional levels during d 1–21 and d 22–42 are presented in Table 1. Feed and fresh water were available *ad libitum*. Birds were vaccinated using combined Newcastle disease virus (**NDV**) and infectious bronchitis virus on d 7 via intranasal and intraocular administration and on d 21 through oral administration.

**Table 1:** Composition and nutrient levels of diets.

Item (% unless noted)	Starter (1–21 d)	Grower (22–42 d)
Ingredients		
Corn (7.8%, crude protein)	57.0	61.0
Soybean meal (43.5%, crude protein)	35.8	31.8
Soybean oil	1.40	2.90
Limestone	1.28	1.01
Sodium chloride	0.35	0.35
Dicalcium phosphate	1.75	1.39
Choline chloride (50%)	0.26	0.20
DL-Methionine (98%)	0.20	0.12
L-Lysine·HCl (99%)	0.05	0.01
Antioxidant	0.02	0.02
Multivitamin*	0.02	0.03
Multimineral^†^	0.20	0.20
Calculated nutrient levels		
Metabolic energy (Mcal/kg)	2.95	3.05
Crude protein	21.0	19.5
Available phosphorus	0.45	0.38
Calcium	1.00	0.80
Lysine	1.20	1.05
Methionine	0.52	0.42

* Supplied per kilogram of diet: retinyl acetate, 9500 IU; cholecalciferol, 2500 IU; α-tocopherol acetate, 30 IU; menadione, 2.65 mg; thiamin, 2 mg; riboflavin, 6 mg; cyanocobalamin, 0.025 mg; biotin, 0.0325 mg; folic acid, 1.25 mg; pantothenic acid, 12 mg; and niacin, 50 mg.

^†^ Supplied per kilogram of diet: copper, 8 mg; zinc, 75 mg; iron, 80 mg; manganese, 100 mg; selenium, 0.15 mg; and iodine, 0.35 mg.

### Sample collection and procedure

Six healthy birds (one from each replicate) were weighed and selected from each treatment according to their average body weight after a 12-h fast at 21 d and 42 d of age. The chicks were necropsied immediately after being anesthetized by an injection of sodium pentobarbital (30 mg/kg body weight) in the wing vein. Then, the length and weight of the small intestine, including the duodenum, jejunum and ileum, and part of the caeca were measured after excision. Samples of the jejunum and ileum (approximately 1 cm obtained from the midpoint) were fixed in 4% paraformaldehyde solution and 2.5% glutaraldehyde solution, respectively, for 24 h for the intestinal histomorphologic indices. Another jejunal and ileal specimen (approximately 10 cm) from the midpoint was opened lengthwise, and digesta samples were harvested and frozen at -20°C for measurements of short-chain fatty acid (SCFA) composition. Meanwhile, the mucosa was gently scraped onto glass slides and similarly stored at -20°C for later determination of antioxidant capacity, and chyme from the caeca was obtained and stored at -80°C until further assay for 16S rDNA sequences.

### Performance measurement

After 8 h of feed deprivation, body weight and feed consumption were recorded for each replicate on d 1, 21 and 42. In this way, the growth performance of broilers was signified by body weight gain (BWG) and feed intake (FI) during each period (d 1–21 and 1–42). Moreover, the feed to gain ratio (F/G) was calculated during the grower period (1–21 d) and the overall period (1–42 d).

### Relative length and weight of intestine

The small intestine and caeca excised from each chick were measured to determine the relative length (cm/kg) and relative weight (g/kg) of intestinal segments, which were reported as the ratio of intestinal tract length (cm) or weight (g) to body weight (kg) based on prior studies by Mahdavi et al. [21] and Jin et al. [24].

### Analysis of intestinal histomorphology

After staining with hematoxylin-eosin, 5-μm consecutive sections of each jejunal and ileal segment were prepared for morphological observations, and images were captured using a LEICA DMi8 optical microscope (Germany). Eight well-oriented villi and the associated crypt of each sample were selected for morphological analysis using Image-Pro Plus (IPP) software. Villus height (VH) was determined from the apex of the villus until the junction of the villus and crypt, and crypt depth (CD) was defined as the depth of invagination between the villus and the basal membrane. The villus height to crypt depth ratio (VCR) was then calculated [17,25]. In addition, eight intact and neat rows of intestinal villi stained with periodic acid Schiff (PAS) were selected to reflect the quantity of goblet cells (GC) between each set of 100 intestinal mucosal epithelial columnar cells (IEL).

Next, samples of jejunum and ileum (d 21) were rinsed three times with 0.1 ml phosphate buffer after being fixed in a mixture of 2.5% glutaraldehyde for at least 24 h at 4°C. The specimens were fixed with 1% osmic acid at 4°C for 2 h, flushed as before and then dehydrated in different gradients (30%, 50%, 70%, 80%, 90%, 100%) of ethanol solutions. They were air-dried using a critical-point drying desiccator (LEICA EM CPD) and secured to stubs mounted with silver tape and sputter coated with gold (EIKO IB-3). The surface morphology and distribution of villi were examined to affirm the effects of sodium butyrate on gut structure and development using a scanning electron microscope (Hitachi S-3400N). Images were photographed at a magnification of 100× and a voltage of 5 kV.

### Antioxidant indices of the intestinal mucosa

The mucosa of the jejunum and ileum was homogenized with nine times the volume of ice-cold physiological saline and centrifuged at 2500 rpm at 4°C for 10 min. The antioxidant enzyme activity of superoxide dismutase (SOD), concentration of malondialdehyde (MDA) and total antioxidant capacity (T-AOC) in 10% homogenate supernatant samples were measured using corresponding commercial kits (Nanjing Jiancheng Bioengineering Institute, Nanjing, P. R. China) according to the manufacturer’s instructions. In addition, the final results of the above indices were normalized by the total protein content of the intestinal mucosa, which was determined using a Pierce® BCA protein assay kit (Thermo Scientific Inc., Waltham, USA).

### Determination of SCFA concentrations

The composition and concentration of SCFAs, acetic acid, propionic acid and n-butyric acid were used as standards and were analyzed by gas chromatography SCION-456-GC with a flame ionization detector. With further modification of the methods described by Hu and Guo [16], approximately 1 g of chyme was dissolved in a mixture of 1% formic acid and 0.1% hydrochloric acid (5 ml), and 0.2 ml 2-ethylbutyric acid (5 mg/ml) was then added. It was then intermittently mixed for 30 min and centrifuged at 12000 rpm for 20 min. The above extract was injected into the column for determination. Nitrogen was used as the carrier gas at a flow rate of 20 ml/min. Furthermore, the hydrogen flow was 30 ml/min, and the air flow was 300 ml/min. Detector and injector temperatures were heated to 230°C and 200°C, respectively. The concentrations of SCFAs were expressed as microgram per gram of chyme sample (μg/g).

### Bacterial 16S rDNA gene analysis of the caeca

The concentration and quality of microbial genomic DNA samples, which were extracted from cecal digesta at 21 d of age with E.Z.N.A® Stool DNA kit (OMEGA, USA) following the manufacturer’s specification, were detected by 1% agarose gel electrophoresis. 16S rDNA sequences spanning the variable regions V3-V4 were amplified with the primer sets of 338 F (5’-ACT CCT ACG GGA GGC AGCAG-3’) and 806 R (5’-GGA CTA CHV GGG TWT CTA AT-3’). Thirty ng of each DNA sample was added for PCR using TransStart Fastpfu DNA Polymerase. PCR procedures were performed as follows: denature at 95°C for 5 min; 30 cycles of 95°C for 30 s, 56°C for 30 s and 40 s at 72°C; extension at 72°C for 10 min; and ending at 4°C. The PCR blended products were examined using 2% agarose gel electrophoresis, purified with Agencourt® AMPure® XP (Beckman Coulter, USA) and eluted with Tris-HCl. Amplicon libraries were sequenced on Illumina MiSeq platform. The library was constructed using NEBNext Ultra II DNA Library Prep Kit (NEB, USA) and detected by Qubit and q-PCR quantification. All the above procedures were conducted by Allwegene Technology Inc. (Beijing, China). Sample reads were assembled by Flash and Pear software, and the clustering of filtered sequences into operational taxonomic units (OTUs) was achieved at a 97% similarity level by Uparse. Analysis of microbial community diversity at different levels (phylum, class, order, family, genus) using RDP Classifier was performed by comparing sequences to the Silva123 database. Beta diversity was visualized by principal component analysis (PCA).

### Statistical analysis

Experimental data were analyzed by one-way ANOVA to examine the effects of dietary treatment using SPSS statistical software (Version 21.0 for Windows, SPSS, Inc., Chicago, IL, USA). Treatment means were tested by Duncan’s multiple comparison when the effects differed significantly. In addition, polynomial regression analysis was also applied to test the linear and quadratic nature of the response to increasing additive doses of sodium butyrate. A probability of *P*<0.05 was considered statistically significant, and 0.05<*P*<0.10 was defined as a tendency towards significance.

## Results

### Growth performance

The effects of dietary sodium butyrate (SB) supplementation on the growth performance of broilers are presented in Table 2. The results indicated that dietary SB supplementation had no significant effects (*P*>0.05) on BWG of chicks, FI or F/G during either the grower period (1–21 d) or the overall period (1–42 d). Likewise, birds receiving a diet supplemented with antibiotics had no apparent changes in FI, BWG or F/G in comparison with the non-supplemented controls (*P*>0.05).

**Table 2:** Effects of sodium butyrate on the growth performance of broilers^1, 2^.

Item	Control	SB1	SB2	SB3	SB4	Antibiotic	SEM^3^	*P*-value		
								Treatment	Linear^4^	Quadratic^4^
1–21 d										
BWG	824	810	803	827	818	801	4.8	0.549	0.763	0.377
FI	1140	1137	1130	1145	1121	1129	4.5	0.710	0.469	0.714
F/G	1.38	1.41	1.41	1.39	1.37	1.41	0.006	0.272	0.230	0.084
1–42 d										
BWG	2445	2437	2427	2401	2422	2496	13.1	0.439	0.435	0.730
FI	4223	4223	4178	4153	4217	4256	17.6	0.640	0.575	0.388
F/G	1.73	1.73	1.72	1.73	1.74	1.71	0.004	0.186	0.396	0.417

^1^ The values are given as the means based on six birds for each treatment (n = 6). Control = basal diet; SB1 = basal diet supplemented with 200 mg/kg sodium butyrate; SB2 = basal diet supplemented with 400 mg/kg sodium butyrate; SB3 = basal diet supplemented with 800 mg/kg sodium butyrate; SB4 = basal diet supplemented with 1000 mg/kg sodium butyrate; Antibiotic = basal diet supplemented with 100 mg/kg aureomycin and 20 mg/kg colistin sulfate.

^2^ BWG, body weight gain (g); FI, feed intake (g); F/G, feed to gain ratio.

^3^ SEM, standard error of the mean.

^4^ Linear and quadratic regression of 5 groups: control and SB1-4.

### Relative length and weight of the intestine

Dietary treatments had significant effects (*P*<0.05) on the relative length of the jejunum, ileum and duodenum at 42 d and on the relative weight of the caeca at 21 d in broilers (Table 3). Specifically, there were quadratic changes (*P*<0.05) in the relative length of the jejunum (at 21 and 42 d) and ileum at 42 d in response to the increasing SB addition, with the SB2 group (400 mg/kg) being the most effective. The SB1 group (200 mg/kg) significantly increased (*P*<0.05) the relative length of the jejunum and ileum at 21 d as well as the duodenum at 42 d when compared with the control group and antibiotics. The relative lengths of the ileum and jejunum at 42 d in the SB2 (400 mg/kg) group were significantly increased (*P*<0.05) compared with those in the control group or antibiotic group. Moreover, the relative weight of the caeca at 21 d in the SB1 group and relative length of the duodenum at 42 d in the SB4 (1000 mg/kg) group demonstrated significant increases compared with those in the control group (*P*<0.05).

**Table 3:** Effects of sodium butyrate on development of the small intestine and caeca in broilers^1^^,^^2^.

Item	Control	SB1	SB2	SB3	SB4	Antibiotic	SEM^3^	*P*-value		
								Treatment	Linear^4^	Quadratic^4^
Relative length										
*d 21*										
Duodenum	29.02	27.98	25.65	26.46	26.02	27.52	0.529	0.421	0.097	0.258
Jejunum	66.90bc	74.74a	68.78b	70.54ab	65.83bc	61.60c	1.006	0.002	0.162	0.026
Ileum	66.84^b^	77.64^a^	66.48^b^	65.52^b^	60.57^b^	61.40^b^	1.408	0.003	0.104	0.112
caeca	13.58	15.32	14.00	12.67	13.78	13.90	0.314	0.296	0.251	0.717
*d 42*										
Duodenum	4.77^b^	5.48^a^	5.22^ab^	4.80^b^	5.75^a^	4.83^b^	0.101	0.007	0.096	0.896
Jejunum	24.97^b^	26.24^b^	29.25^a^	27.10^ab^	24.95^b^	26.00^b^	0.447	0.035	0.965	0.004
Ileum	25.20^ab^	26.65^ab^	28.62^a^	27.07^ab^	23.98^b^	24.07^b^	0.509	0.035	0.380	0.007
caeca	6.08	6.70	6.65	6.32	6.13	6.55	0.103	0.361	0.559	0.050
Relative weight										
*d 21*										
Duodenum	8.05	9.88	9.02	8.83	9.43	8.15	0.266	0.310	0.462	0.446
Jejunum	15.94	17.72	14.57	17.14	17.53	16.96	0.383	0.110	0.217	0.219
Ileum	14.00	14.40	12.67	13.43	12.02	12.02	0.326	0.291	0.094	0.947
caeca	3.62^b^	4.37^a^	3.80^ab^	3.37^b^	3.65^b^	3.53^b^	0.093	0.024	0.074	0.470
*d 42*										
Duodenum	5.26	5.89	5.10	5.29	5.64	5.57	0.107	0.281	0.901	0.545
Jejunum	8.46	9.25	8.46	9.38	8.68	8.36	0.167	0.345	0.643	0.536
Ileum	7.48	7.27	6.86	6.77	6.92	6.75	0.130	0.524	0.154	0.351
caeca	3.25	2.89	3.17	2.91	2.88	2.95	0.069	0.493	0.181	0.861

^a,b,c^ Means in a row with superscripts without a common letter differ, *P*<0.05.

^1^ The values are given as the means based on six birds for each treatment (n = 6). Control = basal diet; SB1 = basal diet supplemented with 200 mg/kg sodium butyrate; SB2 = basal diet supplemented with 400 mg/kg sodium butyrate; SB3 = basal diet supplemented with 800 mg/kg sodium butyrate; SB4 = basal diet supplemented with 1000 mg/kg sodium butyrate; Antibiotic = basal diet supplemented with 100 mg/kg aureomycin and 20 mg/kg colistin sulfate.

^2^ The results are reported as the ratio of the intestinal tract length (cm) or weight (g) to body weight (kg).

^3^ SEM, standard error of the mean.

^4^ Linear and quadratic regression of 5 groups: control and SB1-4.

### Intestinal histomorphologic indices

**Fig 1:**
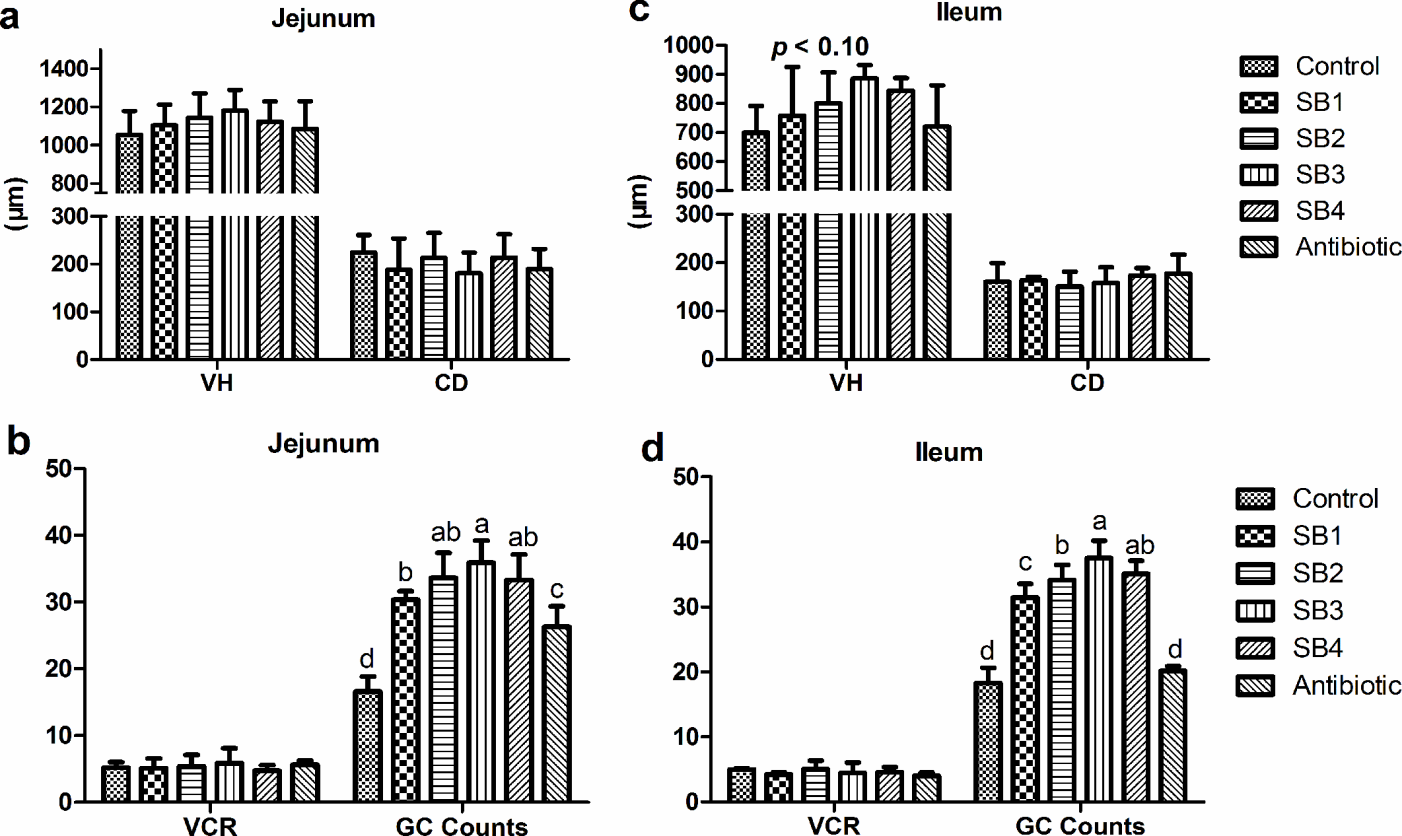
Intestinal histomorphological indices of the jejunum and ileum in broilers. Bars labeled with different letters are significantly different (*P*<0.05), the same as follows. (a, c) VH, villus height (μm); CD, crypt depth (μm); (b, d) VCR, the ratio of VH to CD; GC, goblet cell. Treatments: Control, basal diet; SB1, basal diet supplemented with 200 mg/kg sodium butyrate; SB2, basal diet supplemented with 400 mg/kg sodium butyrate; SB3, basal diet supplemented with 800 mg/kg sodium butyrate; SB4, basal diet supplemented with 1000 mg/kg sodium butyrate; Antibiotic, basal diet supplemented with 100 mg/kg aureomycin and 20 mg/kg colistin sulfate, the same as follows.

**Fig 2:**
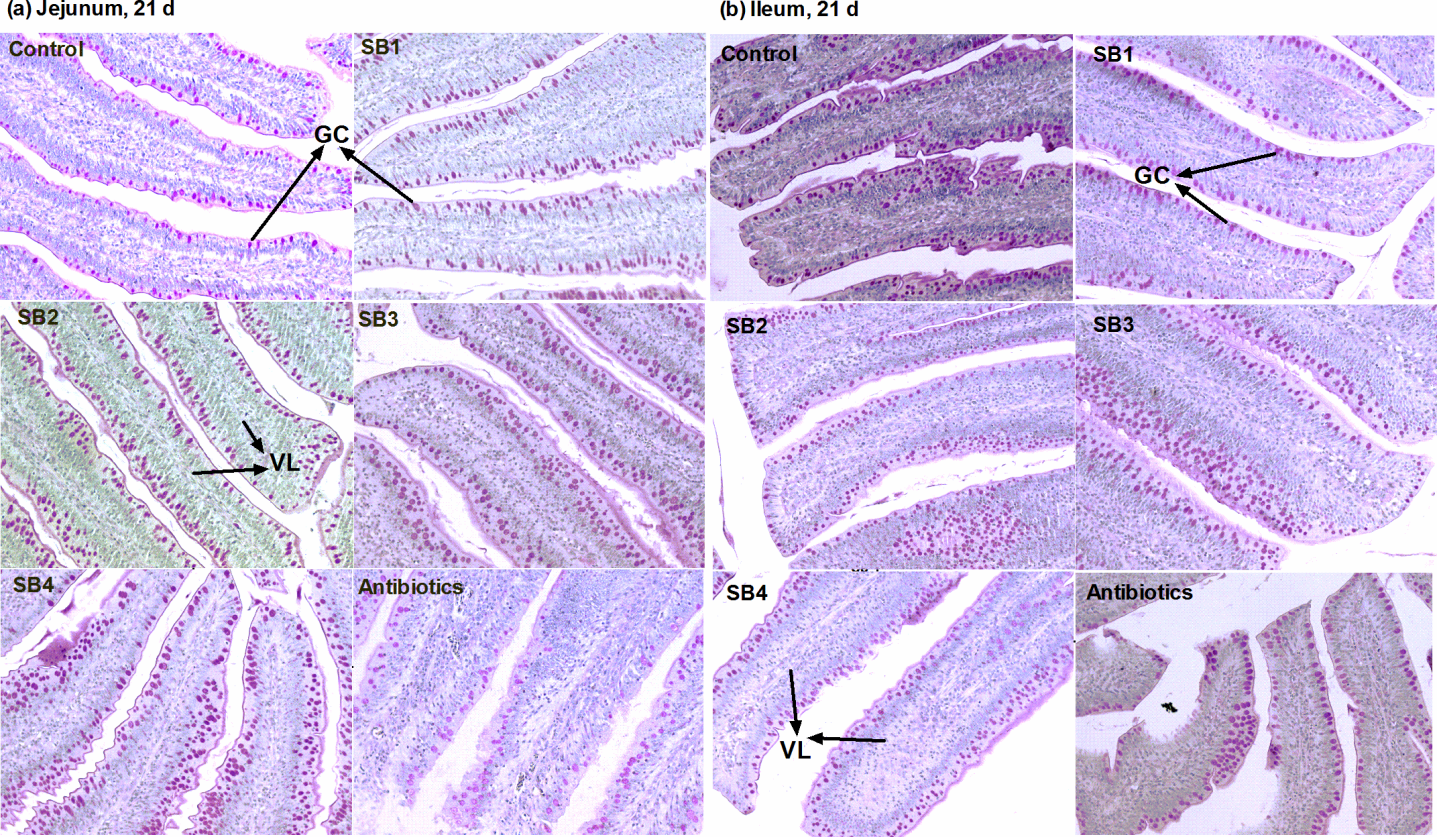
Villus morphology. Jejunum (a) and ileum (b) specimens were collected at 21 d, and PAS-stained sections were analyzed by optical microscopy at 200× for differences in villi (VL) length and quantity of goblet cells (GC).

**Fig 3:**
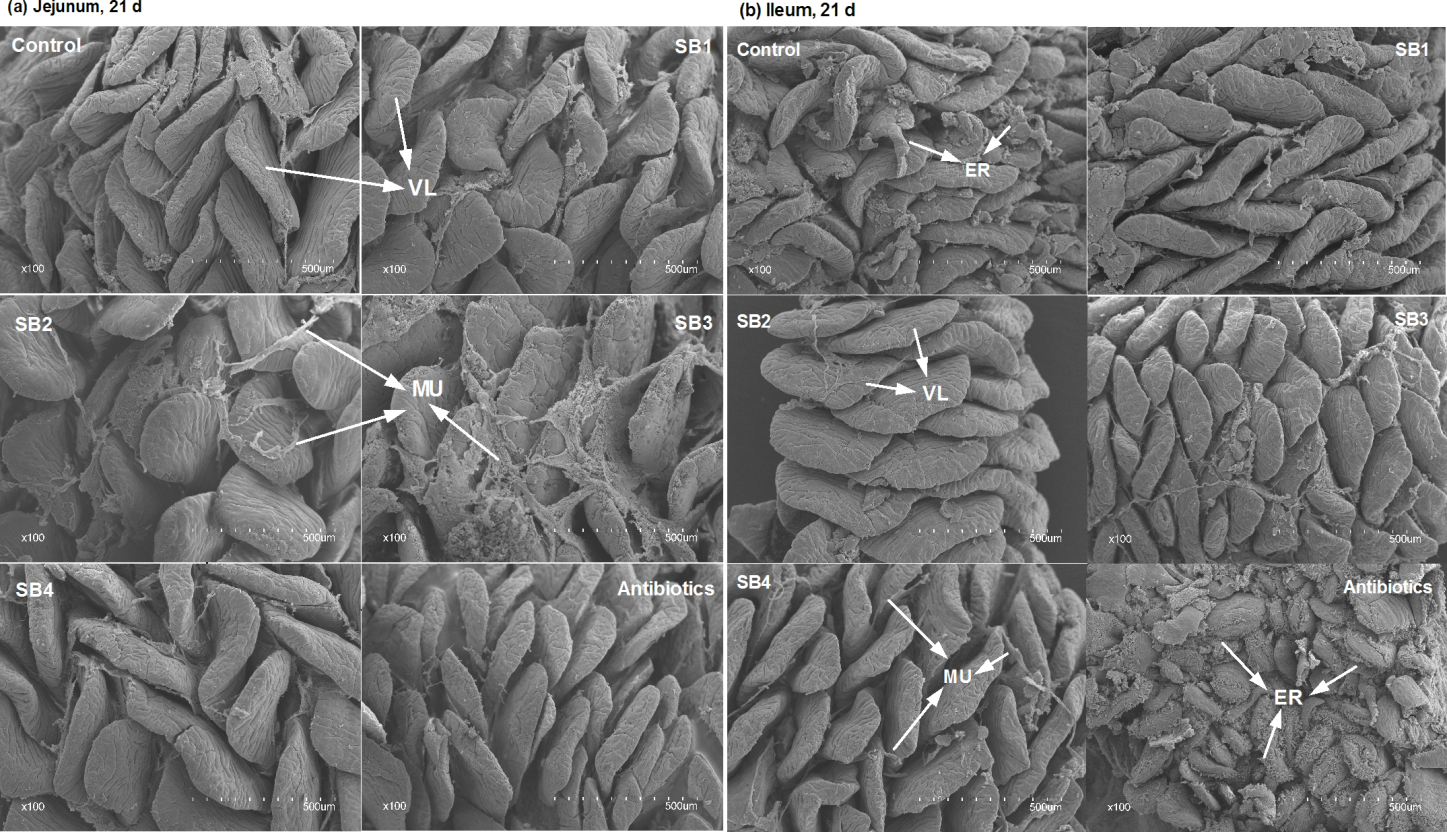
The surface morphology and distribution of villi. Jejunum (a) and ileum (b) specimens were collected at 21 d and then analyzed by scanning electron microscopy at 200× for changes in the appearance and structure of villi (VL) as well as the presence of mucus (MU) or erosion (ER, white arrow).

As indicated in Fig 1, dietary SB supplementation had little relationship (*P*>0.05) with CD coupled with the ratio of villus height to crypt depth (VCR) of the jejunum and ileum. However, the addition of SB tended to increase (*P*_*diets*_<0.10) the ileal VH, with the peak at the medium level (SB3, 800 mg/kg) of experimental diets (Fig 1C), and was also demonstrated to significantly stimulate (*P*<0.05) goblet cells on jejunal and ileal villi, with the medium dose (800 mg/kg) being the most effective (Fig 1B and 1D). We observed that SB-fed broilers showed longer, wider villi and more goblet cells (GC) than the control group or antibiotic group (Fig 2A and 2B). Analysis of the jejunum and ileum by scanning electron microscopy demonstrated more inerratic and flattened leaf-shaped villi (VL) and far fewer erosions (ER) in SB-fed birds (Fig 3A and 3B). In addition, we found more mucus secretion (MU) among VL, which is consistent with the above results related to GC (Figs 1 and 2).

### Antioxidant capacity of the intestinal mucosa

Broilers fed the SB3 (800 mg/kg) diet had greater (*P*<0.05) T-AOC than those in the control group and antibiotic group, while SB significantly decreased (*P*<0.05) the MDA concentration in the jejunal mucosa compared with the control at 21 d of age (Table 4). Moreover, the addition of SB resulted in lower (*P*<0.05) MDA concentrations in the ileal mucosa at 21 d compared with the control, which was similar to the effects of antibiotics. Interestingly, the T-AOC at 21 d of age was significantly reduced (*P*<0.05) in the mucosa of the jejunum of the SB1 group (the low level, 200 mg/kg) in chickens, and SOD of the ileal mucosa of SB4 (the maximum level, 1000 mg/kg) at 42 d was also inferior to the controls (*P*<0.05). Nevertheless, there was no significant increase (*P*>0.05) in antioxidant enzyme activity of SOD among the treatments regardless of age and intestinal segments.

**Table 4:** Effects of sodium butyrate on mucosal antioxidant capacity of the jejunum and ileum in broilers^1^^,^^2^.

Item	Control	SB1	SB2	SB3	SB4	Antibiotic	SEM^3^	*P*-value		
								Treatment	Linear^4^	Quadratic^4^
Jejunum										
*d 21*										
T-AOC	2.08^b^^c^	1.58^d^	1.95^bcd^	2.68^a^	2.20^b^	1.71^cd^	0.078	0.000	0.000	0.556
SOD	212.60	210.77	211.24	195.69	221.79	241.08	7.716	0.665	0.957	0.627
MDA	0.46^a^	0.50^a^	0.49^a^	0.32^b^	0.38^ab^	0.43^ab^	0.019	0.024	0.002	0.467
*d 42*										
T-AOC	2.41	2.43	2.12	1.91	2.11	1.87	0.073	0.109	0.054	0.406
SOD	345.94	368.33	394.36	332.04	311.68	321.28	10.95	0.217	0.104	0.073
MDA	0.98	1.19	1.03	0.85	0.97	1.06	0.074	0.886	0.497	0.784
Ileum										
*d 21*										
T-AOC	2.37	2.59	2.29	2.17	2.10	1.94	0.095	0.504	0.089	0.717
SOD	364.84	326.95	321.57	345.79	313.06	340.99	6.832	0.281	0.182	0.402
MDA	1.35^a^	0.88^bc^	0.95^bc^	0.94^bc^	0.83^c^	1.25^ab^	0.058	0.028	0.023	0.146
*d 42*										
T-AOC	2.77	2.54	2.24	2.69	2.65	2.90	0.076	0.220	0.949	0.113
SOD	323.11^a^	269.76^ab^	305.68^a^	269.03^ab^	208.23^c^	240.51^bc^	9.488	0.002	0.001	0.306
MDA	0.92^ab^	0.79^b^	0.81^b^	0.79^b^	0.83^b^	1.01^a^	0.024	0.038	0.207	0.057

^a-d^ Means in a row with superscripts without a common letter differ, *P*<0.05.

^1^ The values are given as the means based on six birds for each treatment (n = 6). Control = basal diet; SB1 = basal diet supplemented with 200 mg/kg sodium butyrate; SB2 = basal diet supplemented with 400 mg/kg sodium butyrate; SB3 = basal diet supplemented with 800 mg/kg sodium butyrate; SB4 = basal diet supplemented with 1000 mg/kg sodium butyrate; Antibiotic = basal diet supplemented with 100 mg/kg aureomycin and 20 mg/kg colistin sulfate.

^2^ T-AOC, total antioxidant capacity (U/mg prot.); SOD, superoxide dismutase (U/mg prot.); MDA, malondialdehyde (nmol/mg prot.).

^3^ SEM, standard error of the mean.

^4^ Linear and quadratic regression of 5 groups: control and SB1-4.

### SCFA concentrations in intestinal contents

**Fig 4:**
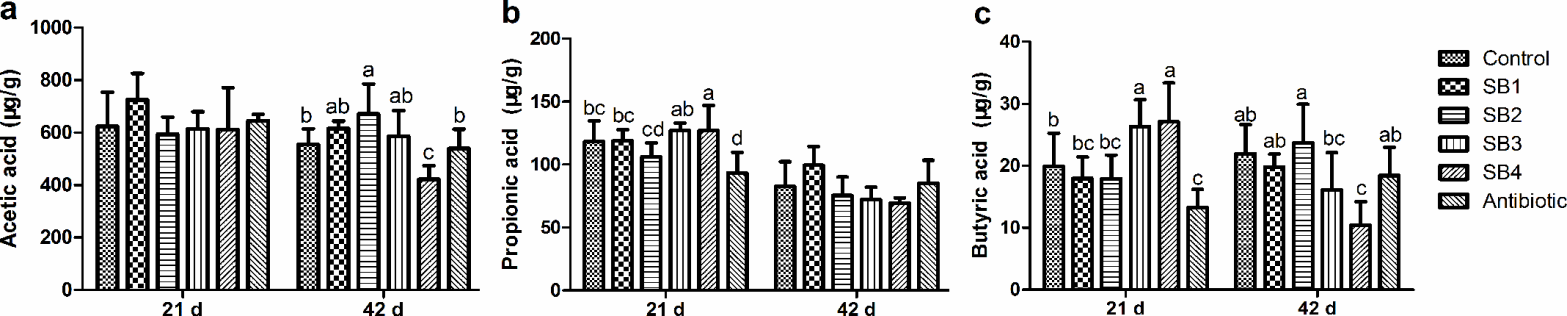
SCFA concentrations in the jejunal chyme of broilers. Concentrations of SCFAs (a, acetic acid; b, propionic acid; c, butyric acid) were expressed as microgram per gram of chyme sample (μg/g).

There were significant differences (*P*<0.05) in several SCFA concentrations in the jejunum chyme among the various treatments (Fig 4). Broilers in the SB4 group (fed SB at 1000 mg/kg) exhibited higher (*P*<0.05) concentrations of propionic acid and butyric acid than those in the control and antibiotic groups at 21 d of age, while lower (*P*<0.05) acetic acid and butyric acid concentrations were found at 42 d of age. Moreover, birds in the SB2 (400 mg/kg) and SB3 (800 mg/kg) groups had higher (*P*<0.05) acetic acid concentrations at 42 d and higher butyric acid concentrations at 21 d, respectively.

**Fig 5:**
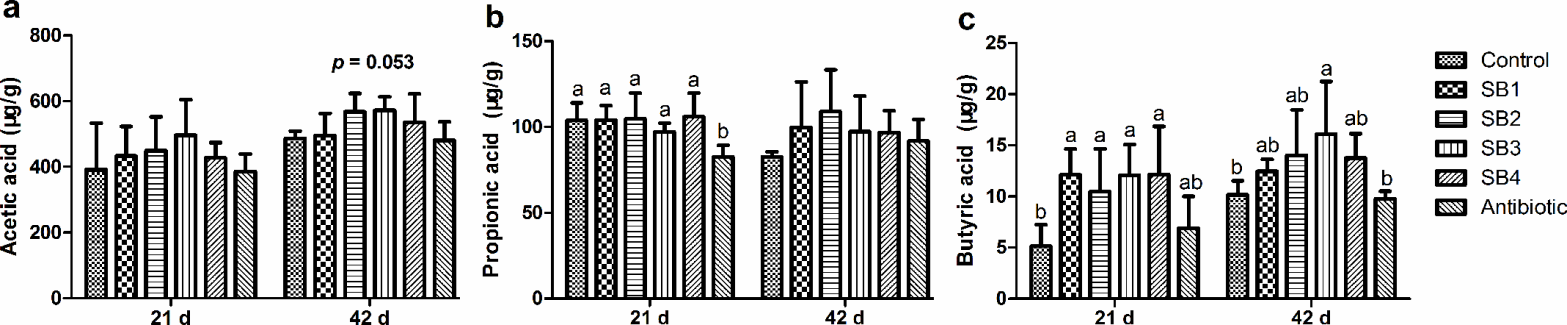
SCFA concentrations in the ileal chyme of broilers. Concentrations of SCFAs (a, acetic acid; b, propionic acid; c, butyric acid) were expressed as microgram per gram of chyme sample (μg/g).

At 21 d of age, birds fed with additional SB had greater (*P*<0.05) butyric acid concentrations in the ileal chyme than the control group, which was similar to the effects of antibiotics (Fig 5). Meanwhile, the supplementation of SB significantly enhanced (*P*<0.05) the concentrations of propionic acid compared to the antibiotic group, whereas no significant differences (*P*>0.05) were detected compared with the control. At 42 d of age, chicks fed SB at 800 mg/kg diet (SB3 group) were found to have higher concentrations of butyric acid (*P*<0.05) in the ileal chyme relative to the control and antibiotic groups, and they simultaneously demonstrated a tendency toward increased (*P*_*diets*_ = 0.053) acetic acid concentrations.

### Microbial community structure of the caeca

**Fig 6:**
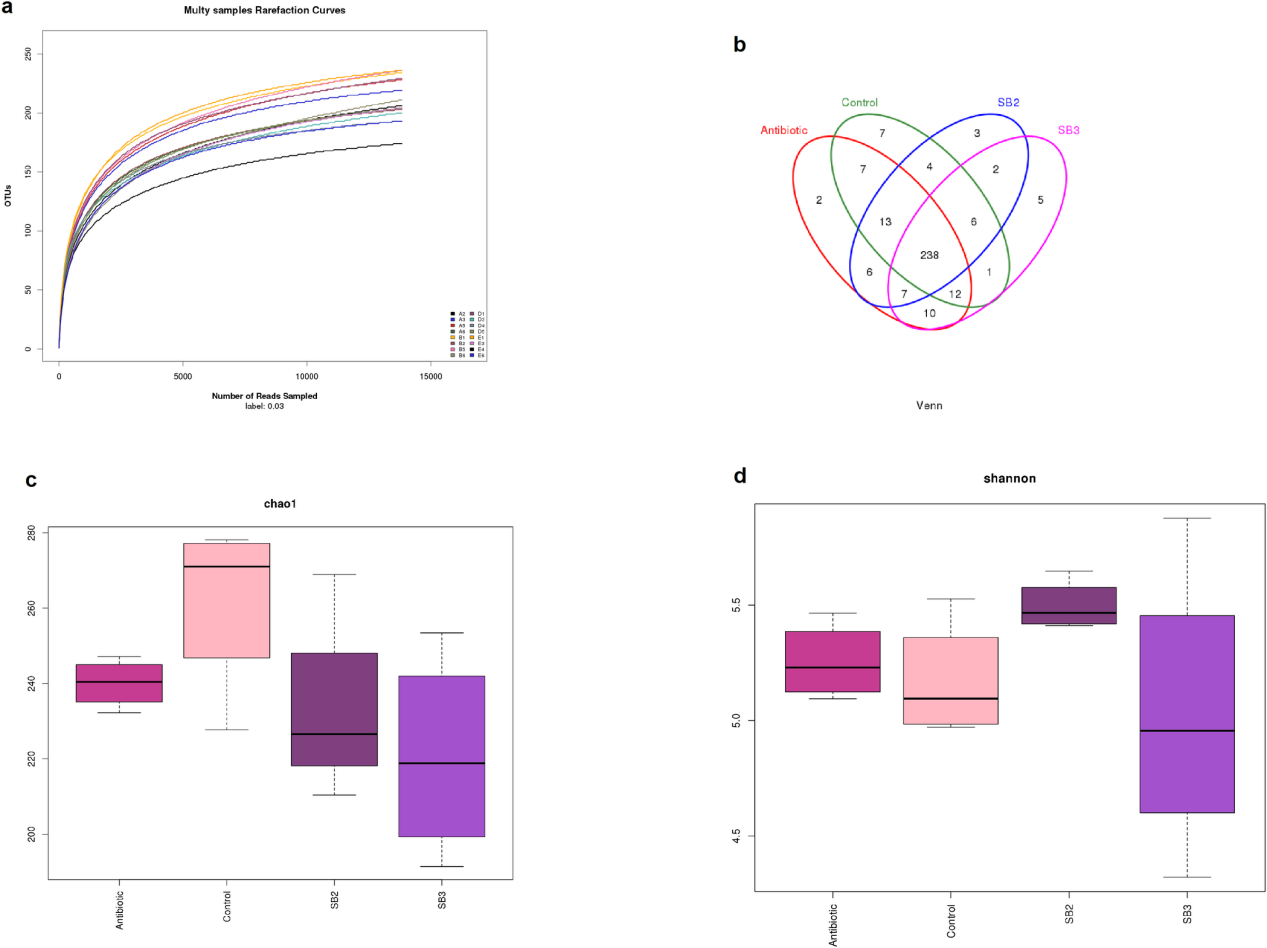
Alpha-diversity of the cecal bacterial community of broilers fed dietary SB supplementation among the 4 treatments (Antibiotic, Control, SB2, SB3). Antibiotic, basal diet supplemented with 100 mg/kg aureomycin and 20 mg/kg colistin sulfate; Control, basal diet; SB2, basal diet supplemented with 400 mg/kg sodium butyrate; SB3, basal diet supplemented with 800 mg/kg sodium butyrate. The same as follows. Cecal chyme from four birds in each group were collected at 21 d for the corresponding analysis. (a) Rarefaction curve for each sample. (b) Venn diagram of the OTUs in the different treatments. (c, d) The bacterial richness estimated by the Chao 1 value and the bacterial diversity estimated by the Shannon index in the caeca.

**Fig 7:**
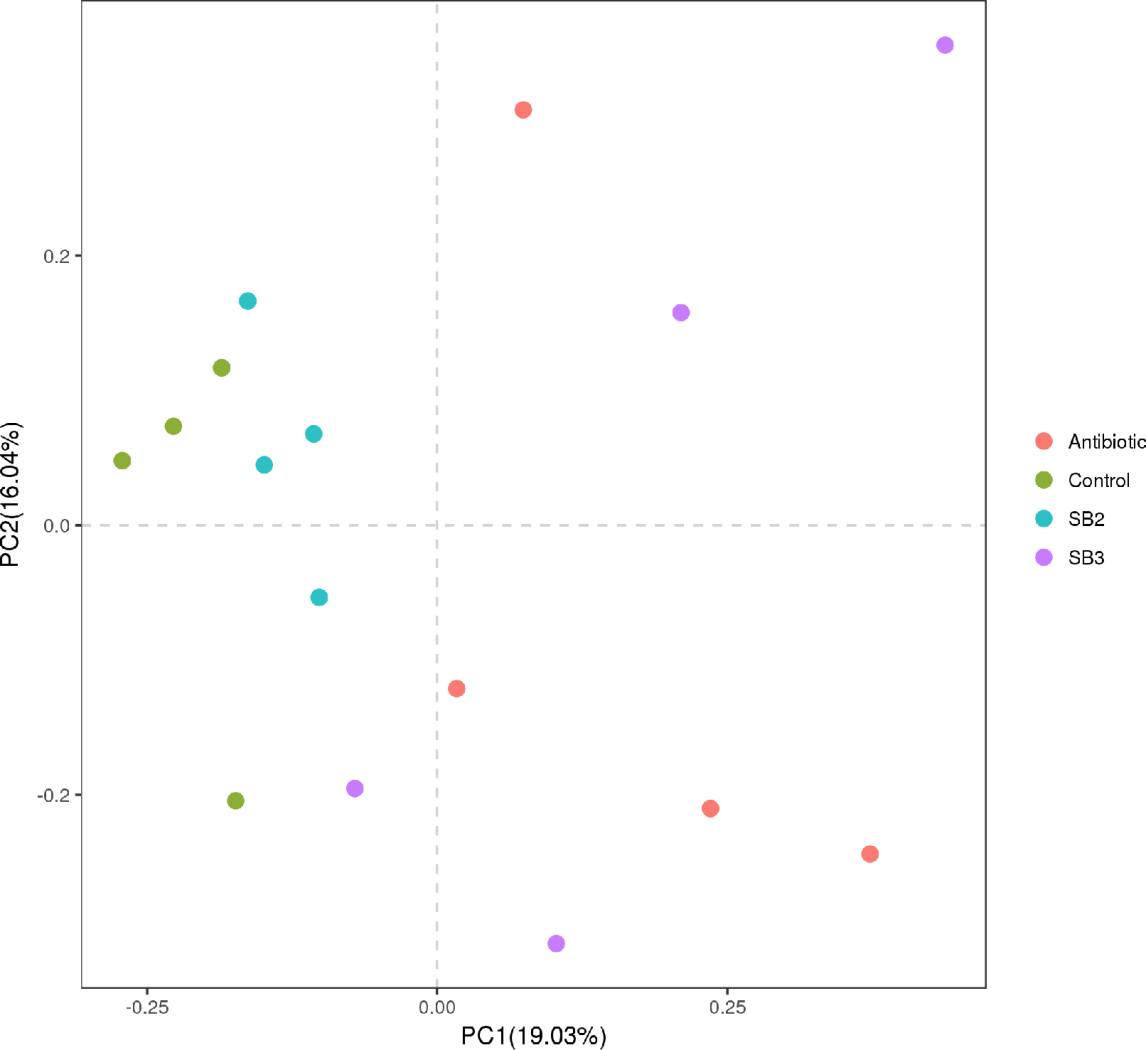
Principal component analysis for cecal microbiota compositions of the 4 treatments (Antibiotic, Control, SB2, SB3) in broilers. Cecal chyme from four birds in each group at 21 d were collected for the corresponding analysis. Abscissa represents the first principal component (PC1), ordinate represents the second principal component (PC2) and the percentages in parentheses represent the contribution value of PC for the sample difference.

In total, 385,158 quality sequences were obtained from bacteria with a read length higher than 250 bp after size filtering, quality control and chimera removal across all 16 samples. The length of the quality sequences accounting for 86.5% was 360–420 bp. All samples approached the saturation plateau based on the rarefaction curves (Fig 6A), suggesting the sampling was sufficient for the majority of the bacterial communities. The cecal bacterial community of four groups shared 238 OTUs and possessed similar amounts of unique OTUs per group, as shown in the Venn diagram (Fig 6B). The Chao value and Shannon index of the bacterial community in the caeca were barely affected by dietary SB supplementation (Fig 6C and 6D, S1 Table). However, within-group differences of the Shannon index in the SB2 group were smaller than the other groups. A PCA was performed to intuitively measure the extent of the similarity between microbiota communities under different treatments. The fecal microbiota from the SB3 and antibiotic groups divided into two intersecting clusters that separated from others and occupied distinct positions in the PCA plot (Fig 7). According to the analysis of molecular variance (AMOVA), the community structures observed in the antibiotics were significantly different from (*P*<0.05) those detected in the control samples (Table 5) and similarly appeared between the SB3 and SB2 groups (*P*<0.05). Therefore, overall, the bacterial microbiota showed a marked divergence among the SB supplementation, antibiotic and control groups (*P*<0.05), and the birds in SB3 shared highly similar gut microbiota with those in the antibiotic group.

**Table 5:** Beta-diversity analysis across broilers fed sodium butyrate or antibiotics*.

Groups compared		AMOVA significance (*P-*value)^†^
Control	SB2	0.156
	SB3	0.072
	Antibiotic	0.025
Antibiotic	SB2	0.062
	SB3	0.696
SB2	SB3	0.027
Global Significance Level		0.010

*Analysis of molecular variance (AMOVA) was used to test if cecal microbial communities were significantly different among treatments (n = 4) at 21 d of age. Control = basal diet; SB2 = basal diet supplemented with 400 mg/kg sodium butyrate; SB3 = basal diet supplemented with 800 mg/kg sodium butyrate; Antibiotic = basal diet supplemented with 100 mg/kg aureomycin and 20 mg/kg colistin sulfate.

^†^ *P*-values returned by Mothur; Significant differences between groups were measured by *P*<0.05.

**Fig 8:**
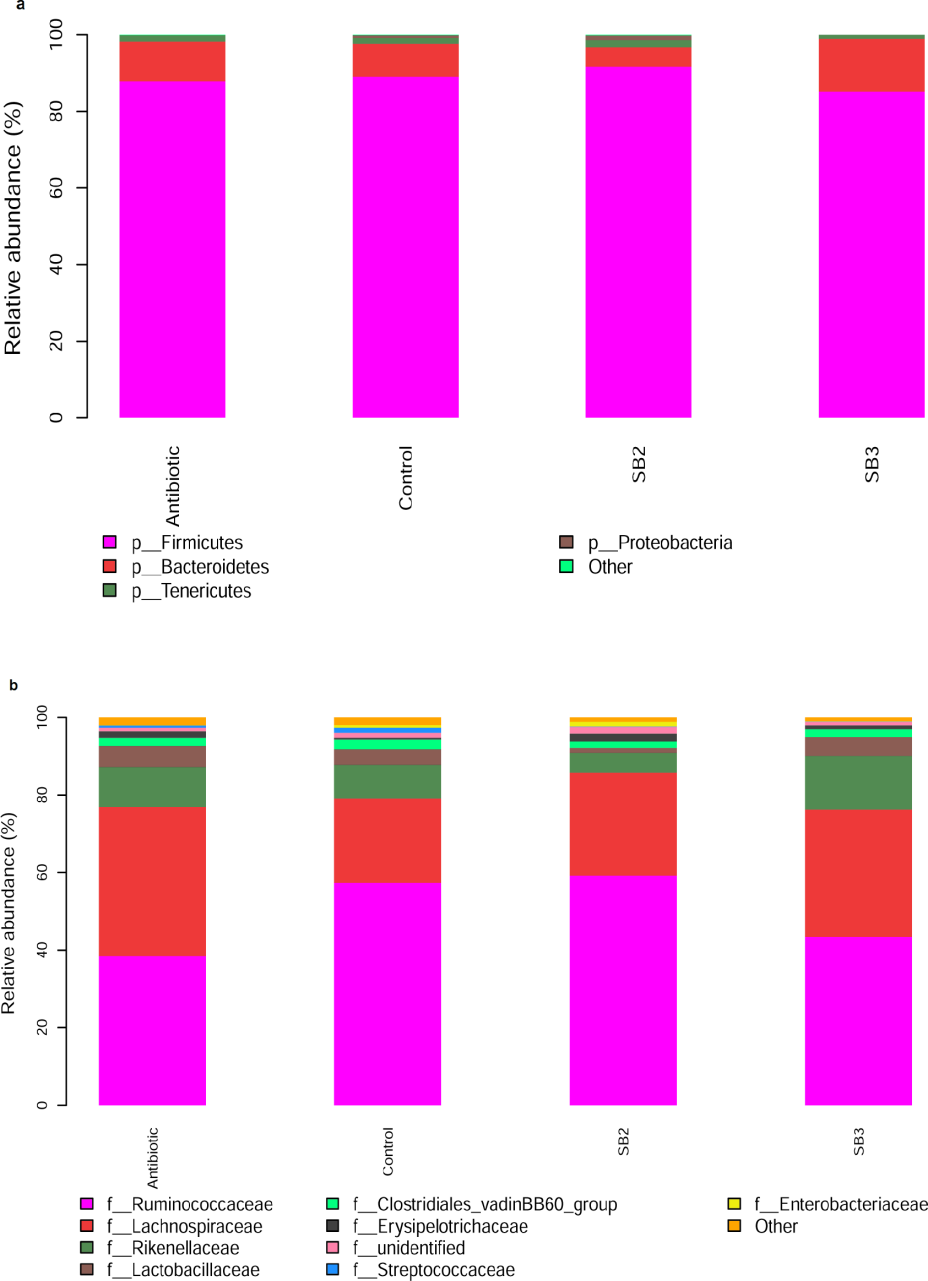
Taxonomic differences in the microbial community of the caeca in broilers. Relative abundance levels of the bacterial phyla (a) and their families (b) are present in the four treatments (Antibiotic, Control, SB2, SB3). Cecal chyme from four birds in each group at 21 d was collected for the corresponding analysis.

The phylogenetic analysis of the bacterial composition in the caeca at 21 d of age is presented in Fig 8. The fecal samples highlighted the predominance of *Firmicutes* followed by *Bacteroidetes*, *Tenericutes* and *Proteobacteria* at the phylum level, together accounting for more than 95% of the total sequences in all groups (Fig 8A). As shown in S2 Table, a noticeable increase in relative abundance (*P*<0.05) was observed in *Bacteroidetes* by the SB3 treatment, from 8.64% in the control birds and 10.39% in the antibiotic group to 13.75% in the SB3 birds. Birds supplemented with the SB2 diet had higher relative abundances of *Firmicutes* (91.64%) and *Proteobacteria* (1.22%), with lower relative abundances of *Bacteroidetes* (5.13%) in comparison to those in the control and antibiotic groups. We also found that decreases in *Proteobacteria* were proportional with exposure to SB3 and antibiotics compared with the control group (*P*<0.05). Fig 8B shows the phylotype distribution of the cecal microbiota at the family level; the majority of classifiable sequences belonged to *Ruminococcaceae* (57.26%), *Lachnospiraceae* (21.72%), *Rikenellaceae* (8.64%), *Lactobacillaceae* (3.96%) and *Clostridiaceae* (2.59%) in the control group, whereas the values for the antibiotic group were 38.58%, 38.33%, 10.39%, 5.36% and 2.12%, respectively (S3 Table). Birds fed with the SB3 addition or antibiotics exhibited a lower proportion of *Enterobacteriaceae* (*P*<0.05) compared to the controls. Apart from the high proportion of *Ruminococcaceae* (59.35%), we also found a noticeable reduction of *Lactobacillaceae* in SB2 broilers compared with the control group (*P*<0.05). In addition, higher proportions of *Lachnospiraceae* and *Rikenellaceae* were observed in the SB3 group.

## Discussion

In the present study, SB addition did not influence the BWG, FI or F/G of broilers under normal feeding management, which is in agreement with the observations of Leeson et al. [20] and Zhang et al. [26]. Conversely, previous reports had indicated that butyrate or its sodium salt (SB, sodium butyrate) mirrors the beneficial effects on growth performance of broilers in terms of increased feed intake coupled with body weight gain and significantly improved FCR [13,16,27]. These variable results may be attributed to the available contents of the SB addition and the type of microbial environment to which the chicks were exposed, as described by Lesson et al. [20] and Smulikowska et al. [15], who detected unremarkable variance in growth performance of healthier broilers raised in an environment with fewer pathogenic bacteria. Moreover, in the present study, specially coated SB additions delivered the portion of butyrate in the further distal intestinal tract because of its slow release during digestion, which caused mucosal modulation in the gut [13,28]. A considerable quantity of butyric acid may therefore be preferentially applied by enterocytes to stimulate intestinal development and function in chicks rather than improve growth performance alone during the starter period. Thus, carry-over effects may affect the overall performance of broilers.

Butyrate, the active ingredient of SB, is readily absorbed by enterocytes as a source of energy [6,21] to accelerate proper intestinal development and function as well as for overall health. Long villi and shallow crypts are generally thought to support a larger surface area for higher absorption capacities and healthy development of the intestine, but they also support greater tissue turnover, thereby accounting for the optimal status of the gut [29–32]. The current study showed that SB tended to increase the ileal VH, although it had no influence on the CD coupled with VCR of the jejunum and ileum, which is similar to previous reports by Smulikowska et al. [15] and Chamba et al. [13]. Moreover, microscopic analysis of the villi also indicated that SB dietary supplements resulted in more goblet cells in both the jejunum and ileum, of which the primary function is the production and stimulation of mucus [33]. The mucus layer, which is the first line of defense in the intestinal mucosa, is composed of large quantities of mucin [34], which is regulated via altering the number of goblet cells or mucin gene expression, particularly MUC2. In support of this observation, we also noted longer, flattened leaf-like villi with far fewer erosions and increased secretion of mucus in chicks supplemented with SB through scanning electron microscopy. In addition, dietary treatments had significant effects on the relative length of the jejunum, ileum and duodenum at 42 d and on the relative weight of the caeca at 21 d in broilers. In brief, the improved intestinal histomorphology and enhancement of goblet cells contributed to the absorption and reinforcement of the intestinal integrity of broilers, which might have resulted in the increased relative weight and length of the small intestine and caeca in our study [17,35].

It has been proposed that oxidative stress due to an imbalance between prooxidant and antioxidant systems caused by stressors [36] contributes to the waste of nutrients as well as to the generation of oxidative damage in living bodies [37]. SOD, a key factor of antioxidant enzymes, plays a role in removing free radicals that arise from MDA, and the level of T-AOC reflects the total antioxidant ability. For instance, dietary CORT addition decreased the antioxidant enzyme activity and increased the MDA concentration, which could be inhibited by SB supplementation in the intestinal mucosa of broilers [22,38]. Similarly, *in vitro* studies concluded that butyrate attenuated H_2_O_2_-induced DNA damage and enhanced CAT activity in human colon cells and the total SOD activity in Caco-2 cells [12,39,40]. In the current study, as expected, broilers fed SB (800 mg/kg, in particular) demonstrated greater T-AOC and decreased MDA concentrations in the jejunal and ileal mucosa at 21 d, which suggested that dietary sodium butyrate enhanced antioxidant properties and retarded damage of the mucosa through scavenging free radicals. It has been hypothesized that butyrate may affect intracellular antioxidants activity, DNA repair systems or (anti)oxidant enzymes to intensify the protection against mucosal oxidative stress [2]. Herein, the improved antioxidant capacity may be associated with gut development and health. Notably, the T-AOC of the jejunal mucosa and SOD of the ileal mucosa were severely inhibited by the minimum and the maximum level of SB, which differed from another study [26]. It is possible, therefore, that an optimal dose of sodium butyrate exhibits protective effects by increasing the level of antioxidants. Aguilar et al. [41] demonstrated that butyrate reduced oxidative stress related to NADPH oxidase down-regulation in the lesion site, by which it was able to attenuate the endothelium dysfunction. However, the definite mechanism by which sodium butyrate mediates antioxidant status in chickens remains to be further investigated.

In the present study, concentrations of SCFAs (mainly acetate, propionate and butyrate) increased markedly to different extents in the jejunum and ileal chyme regardless of age, with higher doses (400, 800 mg/kg) of SB being more effective. This result is in contrast to the findings of Hu and Guo [16], who reported that the absence of any effect of SB on SCFAs concentration was due to absorption in the leading section of the intestine. This discrepancy could be explained by the progressive dissociation and release effects of SB along the length of the gastrointestinal tract as a result of being protected by a special buffer salt system, whereas the latter SB supplementation exerted biological effects in the form of unprotected powder. As Moquet et al. [42] mentioned, unprotected butyrate mainly acted on epithelial cells and microbiota of the crop, proventriculus and gizzard of broilers due to the low pH in the gastric region. Acetate, which is the most abundant SCFA, can be utilized as a precursor for butyrate synthesis and accumulation under conditions accompanied by lactate [43,44]. Propionate has an important impact on the regulation of glucose synthesis and especially gluconeogenesis in many species [45]. Butyrate, an essential source of energy for enteric epithelial cells [6], is quickly absorbed and stimulates growth of the small intestinal epithelium [46]. We can also gain valuable insight into possible gut microbial interactions and microbiota alterations through gut microbe-metabolite associations, which correlate to advantageous effects on host health [47,48]. SCFAs, microbial metabolites fermented from complex carbohydrates, play a major role in the microbiota-host interactions [46]. Thus, we deduced that the enhanced SCFA concentrations, including butyrate in particular, could be essential for appropriate intestinal environment and the accelerated development and health of the gut, which was partially confirmed in our study by the improved intestinal morphological structure and antioxidant capacity of broilers treated with SB diets. Furthermore, this hypothesis pointed towards alterations in intestinal microbiota, as listed below.

A wealth of data indicates that the gut microbiota is a strong determinant of host physiology and health status, particularly its critical role in maintaining the normal physiological function of the intestine [49,50]. A number of previous studies that sought to identify the effects of butyrate or SB addition on microbes in broilers focused on the reduction of specific pathogenic bacteria counts, such as *Escherichia coli*, *Salmonella enteritidis* and *Campylobacter jejuni* [51–53]. Nevertheless, little is known concerning shifts in microbial community and bacterial diversity in the distal intestinal tract of broilers treated with SB using molecular methods. Hence, in the present study, we demonstrated that birds in the SB3 group exhibited distinct gut microbial communities compared to the controls as evidenced by the PCA plot and statistical significance of AMOVA, suggesting that supplemental SB at higher doses (800 mg/kg) influences the intestinal microbiota of broilers. As was reported by Eckburg et al. [54], the hindgut harbors a large number of metabolically active bacteria dominated by the *Firmicutes*, *Bacteroidetes* and *Proteobacteria*, which were related to the fermentation of undigested dietary components [11]. *Bacteroidetes* participates in degrading complex carbohydrates and the synthesis of propionate via the succinate pathway [3]. Likewise, *Firmicutes* are helpful for the polysaccharide decomposition and production of butyrate [3,55]. Similar to a recent study [56], we identified higher *Bacteroidetes* in the SB3 group and higher *Firmicutes* and *Proteobacteria* levels in the SB2 group, which might account for the modulation of gut development and function due to the energy derived from hindgut bacterial fermentation. The *Enterobacteriaceae* family (mainly *Shigella*) is a pathogenic microorganism known for its role in disrupting intestinal digestion and absorption [56]. Thus, the reduction of *Enterobacteriaceae* in birds fed SB3 or antibiotics maintained favorable intestinal environments and health. It has been increasingly identified that a higher level of *Lachnospiraceae* stabilizes the intestinal environment by retarding accumulation of lactate and also produces a certain amount of bacteriocins [57,58]. Meanwhile, the increase in *Ruminococcaceae*, which is connected to cellulos-degrading capacity, could help broilers obtain more energy from complex polysaccharides to promote their intestinal morphological structure. *Lactobacillus* used as a probiotic is beneficial for intestinal health and host growth, but a noticeable reduction of *Lactobacillaceae* was observed in SB2 broilers in our study, which was in accordance with the reports [56]. This unexpected result may be supported and explained by the previous study of De Boever et al. [59], who found that active *Lactobacillus reuteri* excited bacterial bile salt hydrolysis, generating impaired lipid absorption and consequent dietary energy losses. Accordingly, we speculated that a lower proportion of *Lactobacillaceae* may reduce energy losses in birds treated with an SB diet.

## Conclusions

In summary, the present study revealed that dietary SB supplementation promoted intestinal development and health in broilers, as evidenced by the enhanced length and weight of the intestine, increased villus height and increased goblet cells counts, which were accompanied by improvements in antioxidant capacity and SCFA concentrations. Furthermore, SB addition, particularly at higher doses (800 mg/kg), ameliorated the intestinal microbial community and diversity and, as a consequence, might improve the intestinal morphological structure and function of broilers.

## Supporting information

S1 Table Alpha-diversity (Chao1 and Shannon indexes) of the cecal bacterial community of broilers fed dietary SB supplementation among the 4 treatments (Antibiotic, Control, SB2, SB3). Antibiotic, basal diet supplemented with 100 mg/kg aureomycin and 20 mg/kg colistin sulfate; Control, basal diet; SB2, basal diet supplemented with 400 mg/kg sodium butyrate; SB3, basal diet supplemented with 800 mg/kg sodium butyrate. The same as follows.
(DOC)

S2 Table Phylum level microbiota analysis in the caeca of broilers among four treatments (Antibiotic, Control, SB2, SB3). (DOCX)

S3 Table Family level microbiota analysis in the caeca of broilers among four treatments (Antibiotic, Control, SB2, SB3). (DOCX)

## Abbreviations

SB: sodium butyrate
SCFAs: short-chain fatty acids
BWG: body weight gain
FI: feed intake
F/G: feed to gain ratio
VH: villus height
CD: crypt depth
VCR: villus height to crypt depth ratio
PAS: periodic acid Schiff
GC: goblet cells
IEL: intestinal epithelial columnar cells
SOD: superoxide dismutase
MDA: malondialdehyde
T-AOC: total antioxidant capacity
OTUs: operational taxonomic units
PCA: principal component analysis
SEM: standard error of the mean
