# Supplementary material for: Dietary sodium butyrate improves intestinal development and function by modulating the microbial community in broilers
Source: PLoS One. 2018 May 24;13(5):e0197762. doi: 10.1371/journal.pone.0197762 (PMC5967726; doi:10.1371/journal.pone.0197762)
Supplement: S1 Table — Antibiotic, basal diet supplemented with 100 mg/kg aureomycin and 20 mg/kg colistin sulfate; Control, basal diet; SB2, basal diet supplemented with 400 mg/kg sodium butyrate; SB3, basal diet supplemented with 800 mg/kg sodium butyrate. The same as follows. (DOC) [file pone.0197762.s001.doc]

**S1 Table. Alpha-diversity (Chao1 and Shannon indexes) of the cecal bacterial community of broilers fed dietary SB supplementation among the 4 treatments (Antibiotic, Control, SB2, SB3).**

| Alpha diversity | Antibiotic | Control | SB2 | SB3 | *P*-value |
| --- | --- | --- | --- | --- | --- |
| Chao1 | 240.05±6.42 | 261.98±23.46 | 233.11±25.07 | 220.63±27.13 | 0.113 |
| Shannon | 5.25±0.17 | 5.17±0.26 | 5.50±0.11 | 5.03±0.64 | 0.353 |
