# Supplementary material for: Dietary sodium butyrate improves intestinal development and function by modulating the microbial community in broilers
Source: PLoS One. 2018 May 24;13(5):e0197762. doi: 10.1371/journal.pone.0197762 (PMC5967726; doi:10.1371/journal.pone.0197762)
Supplement: S2 Table — (DOCX) [file pone.0197762.s002.docx]

**S2 Table. Phylum level microbiota analysis in the caeca of broilers among four treatments (Antibiotic, Control, SB2, SB3).**

| The phylum (P) level | Antibiotic | Control | SB2 | SB3 |
| --- | --- | --- | --- | --- |
| P_*Firmicutes* | 87.90 | 89.06 | 91.63 | 85.11 |
| P_*Bacteroidetes* | 10.37 | 8.63 | 5.14 | 13.85 |
| P_*Tenericutes* | 1.61 | 1.48 | 1.84 | 0.92 |
| P_*Proteobacteria* | 0.02 | 0.68 | 1.21 | 0.07 |
| P_*Actinobacteria* | 0.09 | 0.08 | 0.17 | 0.03 |
| P_*Cyanobacteria* | 0.01 | 0.07 | 0.02 | 0.01 |
