# Supplementary material for: Dietary sodium butyrate improves intestinal development and function by modulating the microbial community in broilers
Source: PLoS One. 2018 May 24;13(5):e0197762. doi: 10.1371/journal.pone.0197762 (PMC5967726; doi:10.1371/journal.pone.0197762)
Supplement: S3 Table — (DOCX) [file pone.0197762.s003.docx]

| The family (F) level | Antibiotic | Control | SB2 | SB3 |
| --- | --- | --- | --- | --- |
| F_*Lachnospiraceae* | 38.47 | 21.80 | 26.54 | 32.85 |
| F_*Ruminococcaceae* | 38.41 | 57.39 | 59.21 | 43.39 |
| F_*Rikenellaceae* | 10.37 | 8.63 | 5.14 | 13.85 |
| F_*Lactobacillaceae* | 5.36 | 3.95 | 1.23 | 4.92 |
| F_*Clostridiales_vadinBB60_group* | 2.21 | 2.47 | 1.65 | 2.00 |
| F_*Erysipelotrichaceae* | 1.66 | 0.61 | 2.11 | 0.97 |
| F_*unidentified* | 0.91 | 1.24 | 1.71 | 0.92 |
| F_*Anaeroplasmataceae* | 0.80 | 0.39 | 0.24 | 0.11 |
| F_*Streptococcaceae* | 0.56 | 1.29 | 0.10 | 0.07 |
| F_*Christensenellaceae* | 0.46 | 0.46 | 0.20 | 0.25 |
| F_*Defluviitaleaceae* | 0.19 | 0.33 | 0.18 | 0.08 |
| F_*Peptostreptococcaceae* | 0.18 | 0.01 | 0.00 | 0.23 |
| F_*Bacillaceae* | 0.14 | 0.52 | 0.09 | 0.10 |
| F_*Family_XIII* | 0.09 | 0.09 | 0.10 | 0.06 |
| F_*Coriobacteriaceae* | 0.09 | 0.08 | 0.17 | 0.03 |
| F_*Eubacteriaceae* | 0.03 | 0.01 | 0.03 | 0.03 |
| F_*Peptococcaceae* | 0.02 | 0.02 | 0.02 | 0.02 |
| F_*Enterococcaceae* | 0.02 | 0.05 | 0.07 | 0.05 |
| F_*Enterobacteriaceae* | 0.02 | 0.68 | 1.21 | 0.07 |
| F_*Clostridiaceae_1* | 0.00 | 0.00 | 0.01 | 0.00 |

**S3 Table. Family level microbiota analysis in the caeca of broilers among four treatments (Antibiotic, Control, SB2, SB3).**
